# Adiponectin and Leptin at Early Pregnancy: Association to Actual Glucose Disposal and Risk for GDM—A Prospective Cohort Study

**DOI:** 10.1155/2018/5463762

**Published:** 2018-07-15

**Authors:** Latife Bozkurt, Christian S. Göbl, Sabina Baumgartner-Parzer, Anton Luger, Giovanni Pacini, Alexandra Kautzky-Willer

**Affiliations:** ^1^Department of Internal Medicine III, Division of Endocrinology and Metabolism, Unit of Gender Medicine, Medical University of Vienna, Vienna, Austria; ^2^Department of Obstetrics and Gynecology, Division of Feto-Maternal Medicine, Medical University of Vienna, Vienna, Austria; ^3^Metabolic Unit, Institute of Neuroscience, National Research Council, Padova, Italy

## Abstract

**Aim:**

There is scarce information on associations of adipokines, and concurrent glucose disposal during early pregnancy as performance of oral glucose tolerance is uncommon before 24th gestational week. We sought to examine associations of leptin and adiponectin to insulin sensitivity already at early pregnancy before recommended screening for GDM and to describe trajectories of adiponectin in relation to GDM status.

**Methods:**

216 pregnant women were prospectively included at 16th (IQR: 14–18) gestational week (GW) for fasting adiponectin and leptin with subsequent OGTT testing for evaluation of insulin sensitivity and *β*-cell function. Follow-ups of adiponectin were performed at further four visits until 8–12 weeks after delivery.

**Results:**

In early pregnancy, differences in adiponectin and leptin were significant between GDM women (*n* = 82) and controls (*n* = 134), whereby those with early GDM (<21st week, *n* = 49) showed more distinguishing levels (adiponectin: 8.5 ± 3.8 versus 10.4 ± 4.4 *μ*g/ml, *p* = 0.004; leptin 93.4 ± 38.5 versus 78.0 ± 39.2 *μ*g/ml, *p* = 0.005). Both adipokines were significantly associated with insulin sensitivity and *β*-cell function. Their attribution for GDM prediction was moderate to fair and more enhanced in early GDM. Trajectories of adiponectin remained constantly lower in GDM women, whereas dynamics in controls showed initially increased concentrations with decreasing tendency until 3rd trimester. After delivery, low adiponectin was associated with glucose dysregulation.

**Conclusion:**

Associations of adiponectin and leptin with features of deteriorated glucose metabolism at early gestation may be indicative for the endocrine involvement of adipose tissue in the manifestation of GDM and thus predictive for later impairments in metabolic flexibility in women at risk.

## 1. Introduction

Overweight and obesity in women at reproductive age advances the development of insulin resistance and thereby increases risk for early deterioration of pregnancy metabolism [1]. Besides its function as energy storage site, adipose tissue acts as an endocrine organ, secreting proteins that are involved in various physiologic interactions with other organic systems [2]. Among these adipokines, adiponectin and leptin are suggested to play a considerable role in the regulation of whole-body glucose homeostasis.

Although rate of leptin production mainly depends on level of adiposity, the rise in leptin with progressing gestation may be accounted rather for placental production than maternal weight gain [3]. There is much controversial data, but most clinical studies support that during pregnancy, insulin resistance and hyperinsulinemia promote adipocyte leptin synthesis that is independent of maternal BMI and further suggest an association of early hyperleptinemia with later GDM onset [2, 4]. In contrast to leptin, adiponectin is primarily expressed and synthetized in maternal adipose tissue but not by the placenta and furthermore does not cross to fetal circulation [5, 6]. Previous investigations ascribe adiponectin to promote *β*-cell function and survival as well as to lower systemic glucose levels via suppression of hepatic glucose output [7, 8]. There is evidence for an inverse association of serum adiponectin with progressing insulin resistance [7]. This is already corroborated in pregnant populations showing that low adiponectin levels were related to decreasing maternal insulin sensitivity during pregnancy [9]. The applicability of adiponectin as prognostic biomarker for GDM risk is currently under debate as there is evidence, although still limited due to inconsistently applied diagnostic and screening criteria, that decreased adiponectin levels at early pregnancy or even measured 5 years before pregnancy predict later GDM manifestation independent of BMI [4, 10, 11]. However, consideration of baseline insulin sensitivity contemporary to adiponectin measurement performed before recommended GDM screening period could be of interest to better evaluate the predictive value of adiponectin for GDM development.

The aim of this study was to examine the relation of leptin and adiponectin to status of insulin sensitivity at early pregnancy and to evaluate their accuracy for prediction of GDM manifestation. Further, we evaluated characteristics of adiponectin trajectories during the course of gestation until 3-month postpartum between women with GDM and those who remained normal glucose tolerant.

## 2. Materials and Methods

### 2.1. Study Population and Clinical Examinations

This longitudinal study was conducted prospectively at the Department of Internal Medicine III, Division of Endocrinology and Metabolism, Unit of Gender Medicine, Medical University of Vienna, between 2010 and 2014 as previously reported in [12, 13]. Study participants were recruited among pregnant women attending our diabetes and pregnancy outpatient clinics at a tertiary care center ≤ 21st week of gestation (GW, visit 1) for the assessment of glucose tolerance and were reevaluated during further clinical follow-up periods: 24th–28th GW (visit 2), 30th–34th GW (visit 3), and >36th GW (visit 4) as well as 8–12 months after pregnancy (visit 5). Women with known preconceptional diabetes, chronic or serious acute infections, hematological diseases or diseases of the hematopoietic system, severely impaired liver or kidney function, or if they have been tested for hepatitis C antibodies or HIV were not included. All subjects gave written informed consent for participation in the study. The study was approved by the local ethics committee (Ethics Committee of the Medical University of Vienna) and was performed in accordance with the Declaration of Helsinki.

Of the initially 223 patients recruited in the framework of this study, data on adiponectin or leptin was available in 216 pregnant females. All women underwent a broad risk evaluation at the initial assignment including BMI (preconceptional and actual) and obstetric history. For evaluation of glucometabolic status, a 2 h OGTT was performed already during the first visit ≤ 21st week of gestation (median: 16, IQR: 14–18) after a 10–12-hour overnight fast. In case of a negative result, a further clinical evaluation and diagnostic OGTT was performed during the 24–28th weeks of pregnancy according to the IADPSG criteria. Six subjects with negative OGTT results received insulin therapy during follow-up due to incident macrosomia and elevated fasting glucose and thus were classified as GDM. During first assessment within the framework of this study, four women were classified as having preexisting diabetes (i.e., fasting plasma glucose 126 mg/dl, HbA1c > 6.5% (47.54 mmol/mol) at the first antenatal visit) and hence were excluded from further analyses.

### 2.2. Laboratory Methods

Glucose, insulin, and C-peptide were determined from blood samples obtained during the OGTT from blood samples drawn at fasting as well as 30, 60, 90, and 120 min following a 75 g glucose load. All serum parameters were measured according to the international standard laboratory methods at our certified Department of Medical and Chemical Laboratory Diagnostics (http://www.kimcl.at/).

At each visit, an additional venous blood sample was drawn in fasting condition for further laboratory analysis. Herein, total adiponectin and leptin were measured in duplicates using radioimmunoassay (RIA) purchased from Millipore, Billerica, MA [12, 13].

### 2.3. Calculation of Insulin Sensitivity and Secretion

Indices of insulin sensitivity and *β*-cell secretion were calculated from OGTT data: the Matsuda index is validated for estimation of whole-body insulin sensitivity [14]. Insulin resistance in fasting conditions was described by HOMA-IR, approximating of the amount of insulin action in the liver [15]. Total areas under the concentration curves (AUC) of glucose, insulin, and C-peptide were calculated by using the trapezoidal method. Modified insulinogenic indices were calculated to describe early (Δ_Insulin_/Δ_Glucose_ 0–30′), late (AUC_Insulin_/AUC_Glucose_ 60–120′), and total insulin secretion (AUC_Insulin_/AUC_Glucose_ 0–120′) from posthepatic measures [16]. The disposition index (ISSI-2) was calculated as the product of AUC_Insulin_ 0–120′/AUC_Glucose_ 0–120′ and the Matsuda index.

### 2.4. Statistical Analysis

Continuous variables were summarized by means and standard deviations (SD), and categorical variables were summarized by counts and percentages. Comparisons of continuous parameters between two or three groups (cross-sectional) were performed by *t*-test as well as analysis of variance (ANOVA) and Fisher protected least significant for difference tests, respectively. Rank-based procedures were used for comparisons in case of skewed distributed parameters. Differences of categorical variables were assessed by using Fisher's exact test. Odds ratios and 95% confidence intervals (CIs) were computed by binary logistic regression. The predictive accuracy of adiponectin and leptin for GDM manifestation was assessed by binary logistic regression as well as receiver operating characteristic (ROC) analysis, and 95% CIs were estimated by thousand stratified bootstrap replicates. The relationship between metric scaled variables at different visits was assessed by Spearman's rank correlations.

Moreover, linear mixed effects models with random intercepts and random slopes by subjects were used to assess time-dependent changes of parameters of interest (adiponectin) during follow-up in different subgroups, whereby regression coefficients (*β*) represent the mean change in the dependent variable for the increase of one unit (for this analysis, this is the increase per 4 weeks of gestation). A spatial exponential covariance structure was used to model correlations between repeated measurements. A group per time interaction term was included to assess group-specific differences in the slope of trajectories of parameters of interest.

Statistical analysis was performed with R (V3.1.1) and contributing packages (particularly “pROC,” “gmodels,” “nlme,” “lattice,” and “ggplot2”) [17]. A two-sided *p* value ≤ 0.05 was considered statistically significant. There were no considerations to adjust for multiplicity in this report if not otherwise indicated.

## 3. Results

### 3.1. Descriptive Characteristics at First Assessment

**Figure 1:**
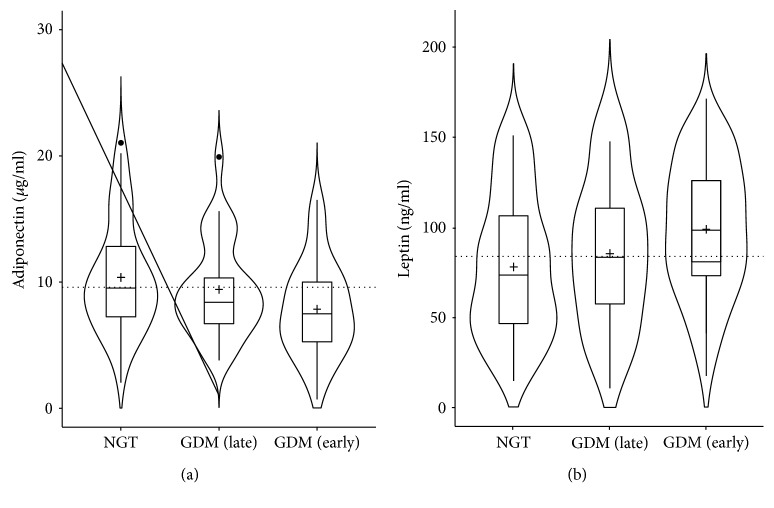
Mean differences in adiponectin (a) and leptin (b) in normal glucose tolerant (NGT) pregnancies and those with early GDM manifestation (<21st week, GDM (early)) and later onset (>24th week, GDM (late)) visualized by violin plots. Figure shows boxplots (inner part of the plot with horizontal lines indicating first, second, i.e., median and third quartile; mean values of the groups are indicated by a cross) and the distribution density (outer part of the plot). The dotted line represents the mean value of the study population at visit 1.

Descriptive characteristics of the study population at first visit (V1) grouped by GDM status are summarized in Table 1. GDM was diagnosed in 82 women, whereby 49 of these were affected by earlier manifestation ≤ 21st GW. 134 women remained normal glucose tolerant (NGT) during pregnancy. Total adiponectin was significantly decreased and leptin more elevated in women with GDM, whereby levels of both adipokines were more distinguishing in women with earlier GDM manifestation compared to NGT (Figure 1, adiponectin: 7.9 ± 3.7 versus 10.4 ± 4.4 *μ*g/ml, *p* = 0.001; leptin 98.9 ± 36.4 versus 78.0 ± 39.2 *μ*g/ml, *p* = 0.002).

### 3.2. Association of Adipokines to Metabolic Parameter

Both adipokines were associated to impaired glucose disposal as estimated by parameters based on 75-OGTT-derived insulin, C-peptide, and glucose measurements at the early antenatal visit: In particular, associations were observable to insulin sensitivity and *β*-cell secretion, respectively, calculated by the Matsuda index (adiponectin: rho = 0.33, 95% CI: 0.18 to 0.45, *p* < 0.001; leptin: rho = −0.48, 95% CI: −0.59 to −0.36, *p* < 0.001), HOMA-IR (adiponectin: rho = −0.25, 95% CI: −0.39 to −0.11, *p* < 0.001; leptin: rho = 0.42, 95% CI: 0.29 to 0.54, *p* < 0.001), ISSI-2 (adiponectin: rho = 0.26, 95% CI: 0.10 to 0.40, *p* = 0.001; leptin: rho = −0.31, 95% CI: −0.43 to −0.17, *p* < 0.001), and preconceptional BMI (adiponectin: rho = −0.37, 95% CI: −0.50 to −0.22, *p* < 0.001; leptin: rho = 0.74, 95% CI: 0.66 to 0.80, *p* < 0.001)

### 3.3. Predictive Accuracy of Adipokines for GDM

**Figure 2:**
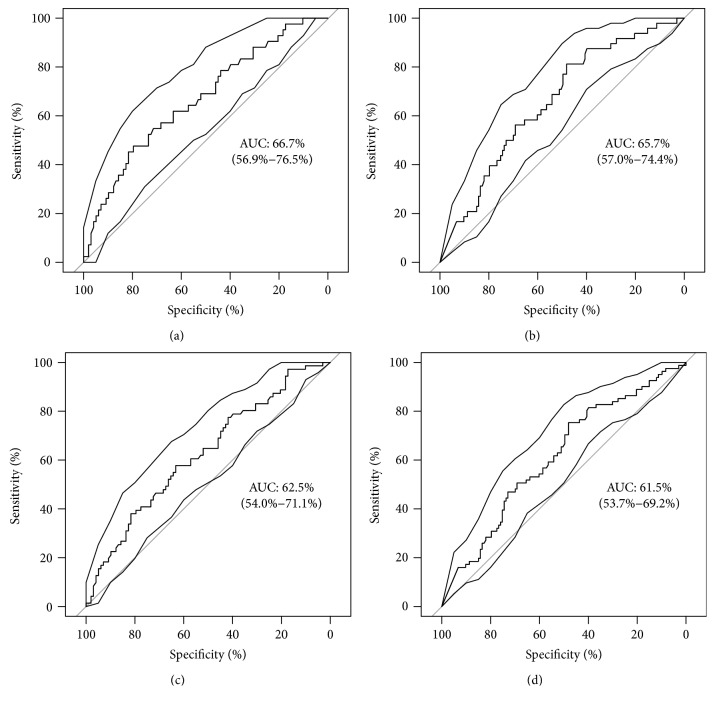
ROC curves for baseline adiponectin and leptin for GDM prediction grouped by time of GDM onset: (a) adiponectin in early manifested women, (b) adiponectin in late manifested women, (c) leptin in early manifested women, and (d) leptin in late manifested women.

Both adipokines in early pregnancy < 21st GW were moderate to fair predictive for GDM manifestation, whereby this association was more enhanced in those with early onset < 21st GW as shown in Figure 2 (adiponectin: 0.67, 95% CI: 0.57 to 0.77; leptin: 0.66, 95% CI: 0.57 to 0.74). The predictive accuracy was not substantially improved when the information of both adipokines was combined by logistic regression (ROC statistics for early onset: 0.69; ROC statistics for late onset: 0.64). The ROC statistics of preconceptional BMI were 0.72 (95% CI: 0.64 to 0.80) and 0.66 (95% CI: 0.59 to 0.73) for early and late onset of GDM, respectively. Logistic regression revealed that in contrast to leptin (*p* = 0.670), the association of adiponectin with early GDM manifestation was independent of preconceptional BMI (*p* = 0.034).

### 3.4. Trajectories of Adiponectin

**Figure 3:**
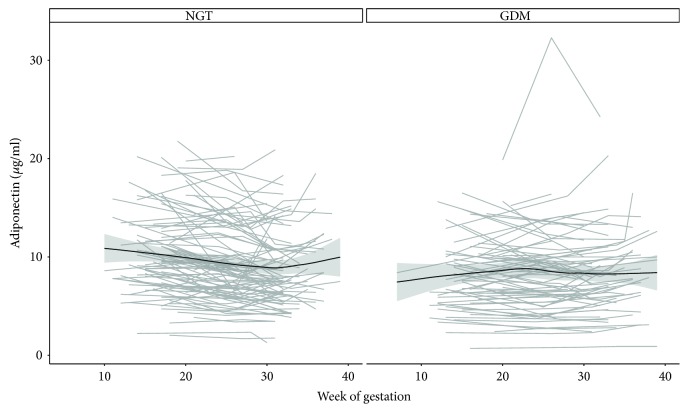
Trajectories of serum adiponectin during pregnancy in normal glucose tolerant (NGT) women as well as women with gestational diabetes mellitus (GDM). Dashed line represents the 50% quantile (i.e., the median) of all measurements during gestation. Solid line represents a trend curve derived by locally weighted regression.

Serum levels of adiponectin constantly remained at lower levels from beginning to end of pregnancy in women affected by GDM. In contrast, pregnancies with normal glucose tolerance showed higher levels at baseline examination followed by a consistent decrease during advancing pregnancy (decreased by 0.25 *μ*g/ml per 4 weeks) resulting in a significant group-by-time interaction (*β*: 0.26, 95% CI: 0.13 to 0.38, *p* < 0.001). However, it has to be mentioned that the trajectories in the NGT group progressed into a slight increase shortly before delivery as notable in Figure 3 showing the trajectories for adiponectin for both groups. Our basic conclusions about the specific patterns of adiponectin remained constant after accounting for dynamic changes in weight during pregnancy.

### 3.5. Postpartum Associations of Adiponectin and Glucose Metabolism

At reexamination 3–12-month postpartum (*n* = 117; GDM: 55, NGT: 62), there was no difference in adiponectin between women with prior GDM compared to controls. However, there was a correlation of adiponectin to fasting (rho = −0.29, 95% CI: −0.46 to −0.10) as well as 60 min (rho = −0.39, 95% CI: −0.58 to −0.18) and 120 postload glucose levels (rho = −0.42, 95% CI: −0.58 to −0.23). Upon these results, we reevaluated our data under consideration of postpartum glucose status and observed significant lower levels of adiponectin in women with deteriorated glucose tolerance compared to those with normal glucose tolerance after prior GDM (6.9 ± 3.4 versus 10.0 ± 4.3, *p* = 0.008) as well as to controls (6.9 ± 3.4 versus 9.0 ± 4.3 *μ*g/ml, *p* = 0.038).

## 4. Discussion

Pregnancy per se is a condition affected by metabolic rearrangements of adipose tissue that are physiologically mediated by progressive insulin resistance with advancing gestation [17]. The link between hypoadiponectinemia and reduced insulin sensitivity is apparent as adiponectin levels show a decrease towards end of gestation in healthy women [18]. Also, further studies consistently reported lower circulating levels of adiponectin in GDM compared to non-GDM pregnancies even considering the differing time points of measurements in the respective populations [2, 4, 10]. For our knowledge, this is the first study performing a 75 g glucose tolerance test for estimation of measures of insulin sensitivity and *β*-cell function simultaneously with determination of systemic adiponectin and leptin concentrations at early pregnancy. The degree of glycemic deterioration seems to impact circulating adiponectin levels, as these were more decreased in women with earlier onset of GDM and further were associated to parameters indicative for impaired whole-body insulin sensitivity as well as *β*-cell dysfunction already at early gestational period. Although the secretion of adiponectin is adipocyte specific, there is evidence that rather fat quality than fat mass is the main force for its expression [19]. Indeed, investigations demonstrated higher adiponectin levels in metabolic healthy than unhealthy obese individuals [20, 21]. Given the close relationship between adiponectin and insulin sensitivity, adiponectin might display adipose tissue health and thus influence systemic metabolic flexibility in a wider range as assumed. Corroborating our results, Retnakaran et al. showed in a cross-sectional examination by using measurements of a 100 g OGTT (after an abnormal 50 g testing) that adiponectin in late pregnancy is independently correlated to *β*-cell dysfunction [22]. Lacroix et al. described lower adiponectin concentrations during first and second trimesters to be associated with insulin resistance estimated by an OGTT at 24th–28th gestational weeks. Low adiponectin in the first trimester related to higher risk of developing GDM [23]. Our data confirm and expand these previous results by additionally showing a simultaneous association of adiponectin and OGTT-derived indices already at early pregnancy period.

On the other side, current data on leptin in association to GDM is more controversial. This may be due to the ubiquitous relation of leptin to increasing adiposity, possibly a reflection of leptin resistance that seems to impact dysglycemia [24, 25]. However, some investigations found circulating leptin to be independently associated to GDM prevalence during pregnancy [4]. Our data supports that early gestational leptin is associated to state of insulin resistance and *β*-cell function. There is much debate about the direction of causality between altered adipokines and insulin resistance status in GDM. However, adiponectin and leptin levels at early pregnancy seem to provide a fair reflection of actual whole-body insulin sensitivity and may enhance identification of women at risk.

Actually, two recent systematic reviews independently reported that GDM development was associated with an earlier decrease in adiponectin [4, 10]. However, the quoted summary prognostic accuracy was only at moderate level [10]. Likewise, for leptin, an increased level of 7.25 ng/ml was indicated in a meta-analysis as pooled mean difference in women before GDM manifestation compared to those remaining normal glucose tolerant [4]. Our data support that the overall predictive accuracy of both adiponectin and leptin is only moderate. However, we found that the predictive capacity of both adipokines was more enhanced in recognizing cases with earlier GDM manifestation. It is further noteworthy that adiponectin operated in this term even independent of BMI. These data support the theory of chronic pregestational insulin resistance to be rather the precursor or even cause of more defective adipokine regulation in young women. It is assumed that these circumstances consequently further promote *β*-cell dysfunction and hence determine manifestation time and severity of GDM during pregnancy. Accordingly, lower levels of adiponectin measured 6 years prior to pregnancy were associated with a fivefold increased risk of GDM manifestation in a nested, case-control study by Hedderson et al. [11].

Another advantage of adiponectin's usage as biomarker is its rather independent production from the influencing factors related to growing endocrine activity of the placenta during pregnancy progress [6]. Recently, Haghiac et al. ruled out any expression or de novo production by the placenta and indicated maternal adipose tissue as the main source of adiponectin [5]. Thus, it is to be assumed that changes in whole-body insulin sensitivity are more appropriately mirrored by dynamic adiponectin patterns occurring throughout gestation as corroborated by former studies in normal glucose tolerant women [18]. Similar patterns were also observed in the control group of our study population, but women with gestational diabetes showed constantly low levels during pregnancy reflecting the profound deterioration in glucose metabolism.

After pregnancy, the declining capability of the *β*-cells to compensate growing systemic demands on the background of chronic increase of insulin resistance makes these women prone to manifestation of type 2 diabetes in the years thereafter [1, 26–28]. Hypoadiponectemia is regarded as another indicator of disease progression as follow-up examinations of women with history of GDM showed decreased adiponectin early after pregnancy that is linked to risk for type 2 diabetes, fatty liver, and cardiovascular disease [26]. In accordance in our longitudinal study, lower levels of adiponectin were continuingly observed in women, who in contrast to those regaining their regular metabolism exhibited enduring impairment in glucose tolerance subsequently after pregnancy with GDM. In contrast to other studies, longitudinal analyses have to deal with a considerable limitation due to numbers of loss to follow-up after pregnancy as young mothers tend to neglect their metabolic risk after delivery—a general problem of adherence in clinical routine resulting in discontinuity of care in these women [29]. However, available data support the plausibility of adiponectin as prognostic biomarker that seems to abundantly capture the whole entity of the GDM phenotype from pregnancy to postpartum.

In summary, circulating levels of adiponectin and leptin are related to simultaneous insulin sensitivity at early pregnancy and may impact time of GDM manifestation as those with early onset < 21st gestational week showed distinguishing concentrations compared to normal glucose tolerant pregnancies, although the overall predictive values of both were only moderate to fair. During pregnancy progress, GDM women showed constantly lower concentrations of adiponectin in contrast to unaffected pregnancies as those women were characterized by decreasing trajectories towards end of gestation of the initially increased levels. Moreover, adiponectin remained decreased in those with deteriorated glucose tolerance at early postpartum after pregnancy with GDM. Thus, we emphasize that since pregnancy is a unique situation provoking a condition of prediabetic state particularly in women prone to diabetes and adiposity, derangements of adiponectin and leptin could have implications in the manifestation of GDM—in this context, quality of fat may be an important aspect for further investigations.

## Figures and Tables

**Table 1:** Characteristics and metabolic parameters at first assessment in normal glucose tolerant (NGT) pregnancies and women manifesting gestational diabetes mellitus (GDM).

	*n*	GDM	*N*	NGT	*p* value
Age (years)	82	33.32 ± 4.5	138	31.51 ± 5.4	0.011
Gestational week of first testing	82	15.0 (13–18)	138	16.0 (14–18)	0.111
BMI at first visit (kg/m^2^)	82	30.31 ± 6.2	138	27.14 ± 5.3	<0.001
Pregestational BMI (kg/m^2^)	82	28.79 ± 5.7	138	25.68 ± 5.6	<0.001
FPG (mg/dl)	81	85.5 ± 10.4	138	77.85 ± 5.8	<0.001
Insulin (*μ*U/ml)	81	4.91 (1.90–9.37)	137	3.64 (1.90–7.77)	0.248
C-peptide (ng/ml)	81	1.99 ± 1.37	137	1.53 ± 0.67	<0.001
HOMA	81	0.96 (0.44–2.00)	137	0.71 (0.37–1.47)	0.02884
Matsuda index	78	7.67 ± 4.72	121	10.16 ± 5.83	0.002
ISSI-2	78	2.32 ± 0.98	121	3.56 ± 1.28	<0.001
AUC_Insulin_/AUC_Glucose_	78	33.7 (21.9–49.9)	121	37.5 (26.9–58.6)	0.105
IGI	80	53.9 (30.4–80.8)	128	84.8 (53.5–144.3)	<0.001
Leptin (ng/ml)	81	93.4 ± 38.5	133	78.0 ± 39.2	0.005
Adiponectin (*μ*g/ml)	71	8.50 ± 3.79	98	10.37 ± 4.39	0.004

Data represent means and standard deviations as well as medians and IQR. BMI: body mass index; FPG: fasting plasma glucose; HOMA: homeostasis model assessment; ISSI 2: disposition index; area under the curve for glucose and insulin, respectively, measured during OGTT: AUC_Glucose_ and AUC_Insulin_; IGI: insulinogenic index.

## Data Availability

The data used to support the findings of this study are available from the corresponding author upon request.

## Abbreviations

AUC: Area under the concentration curves
ANOVA: Analysis of variance
BMI: Body mass index
CI: Confidence interval
ISSI-2: Disposition index
FPG: Fasting plasma glucose
GDM: Gestational diabetes mellitus
GW: Gestational week
HOMA: Homeostasis model assessment
IGI: Insulinogenic index
NGT: Normal glucose tolerance
OGTT: Oral glucose tolerance test
RIA: Radioimmunoassay
ROC: Receiver operating characteristic
SD: Standard deviation.
